# Supplementary material for: Isolation and Characterization of Two Novel Siphoviruses Novomoskovsk and Bolokhovo, Encoding Polysaccharide Depolymerases Active against Bacillus pumilus
Source: Int J Mol Sci. 2022 Oct 26;23(21):12988. doi: 10.3390/ijms232112988 (PMC9658697; doi:10.3390/ijms232112988)
Supplement: Supplementary file 1 [file ijms-23-12988-s001.zip › ijms-1869682-supplementary.pdf]

Article

Isolation and Characterization of Two Novel Siphoviruses Novomoskovsk And Bolokhovo Encoding Polysaccharide Depolymerases Active Against Bacillus pumilus

Anna V. Skorynina[1]*, Olga N. Koposova[1], Olesya A. Kazantseva[1], Emma G. Piligrimova[1], Natalya A. Ryabova[2] and Andrey M. Shadrin[1]*

1 G.K. Skryabin Institute of Biochemistry and Physiology of Microorganisms, Pushchino Scientific Center for Biological Research of the Russian Academy of Sciences, Federal Research Center, Pushchino, Prospect Nauki, 5, Russia

2 Institute of Protein Research RAS, Pushchino, Institutskaya st., 4, Russia

* Correspondence: Anna V. Skorynina, Phone: +7(953)967-44-37. E-mail: s_an.net@mail.ru; Andrey M Shadrin, E-mail: andrey2010s@gmail.com.

Supplementary Information:

Table S1. Annotation of the *Bacillus* phage Bolokhovo

ORF№	Start codon	Stop codon	Strand	Blast results: Name	Blast results: E-val	Conserved domains, Blast: Name, (region)	Conserved domains, Blast: E-val	Hhpred results (Prob./E-val)	Annotation
1	65	739	+	putative terminase small subunit [*Bacillus* phage 056SW001B]	$1e^{-77}$			sm_term_P27; phage terminase, small subunit, putative, P27 family (89,28/3,6)	putative terminase small subunit
				putative terminase small subunit [*Bacillus* phage 019DV002]	$6e^{-76}$				
				putative terminase small subunit [*Bacillus* phage 276BB001]	$6e^{-76}$			Terminase_4; Phage terminase, small subunit (88,04/5,6)	
				exopolyphosphatase domain protein [*Geobacillus* phage GBK1]	$3e^{-64}$			COG3747; Phage terminase, small subunit [Mobilome: prophages, transposons], (86,89/9,2)	
2	858	1187	+	hp					hp
3	1533	1252	-	hp				P2_Phage_GpR; P2 phage tail completion protein R (GpR) (90,17/1,7)	hp
4	1802	2083	+	hp				hp	hp
5	2412	2134	-	hp				hp	hp
6	2554	2399	-	hp				PMP1_2; ATPase proteolipid family (97,25/0,002)	Predicted lipoprotein signal peptide
								Mucin15; Cell-membrane associated Mucin15 (97,22/0,00073)	

								PRIMA1; Proline-rich membrane anchor (1 96,09/0,015)	
								STEVOR; variant surface antigen, stevor family, (95,68/0,058)	
7	2968	2633	-	hp				hp	hp
8	3304	3047	-	hp				hp	hp
9	3408	5093	+	terminase large subunit [*Bacillus* virus Glittering]	0.0	Terminase_GpA (52-314)	$6.85e^{-06}$	Terminase_GpA; Phage terminase large subunit (GpA) (100/$6,4e^{-32}$)	Terminase large subunit
				terminase large subunit [*Bacillus* virus Riggi]	0.0			Terminase_1; Phage Terminase (100/$1,2e^{-30}$)	
				terminase large subunit [*Bacillus* virus Blastoid]	0.0			YbcX; Phage terminase, large subunit GpA (99,98/$4,1e^{-28}$)	
10	5175	6494	+	portal protein [*Bacillus* virus Curly]	0.0	DUF935 (57-394)	$7.58e^{-21}$	gp29; Mu-like prophage protein gp29 (100/$6,1e^{-30}$)	portal protein
				portal protein [*Bacillus* virus Glittering]	0.0	gp29 (92-413)	$4.39e^{-09}$	portal_HK97; phage portal protein, HK97 family (99,97/$2,5e^{-28}$)	
				portal protein [*Bacillus* phage Leo2]	0.0			BeeE; Phage portal protein BeeE (99,97/$1,1e^{-28}$)	
11	6548	7450	+	phage head morphogenesis protein [*Bacillus* virus Curly]	0.0	phageSPP1_gp7 (150-261)	$9.02e^{-16}$	COG2369; Uncharacterized conserved protein, contains phage Mu gpF-like domain (99,87/$2e^{-19}$)	Minor head protein
				minor head protein [*Bacillus* virus Glittering]	0.0	Phage_Mu_F (144-254)	$9.57e^{-15}$	phageSPP1_gp7; phage putative head morphogenesis protein, SPP1 gp7 family (99,83/$1,2e^{-19}$)	
				minor head protein [*Bacillus* phage Leo2]	0.0	COG2369 (150-261)	$1.50e^{-04}$	COG5585; NAD+--asparagine ADP-ribosyltransferase (99,72/$1,3e^{-15}$)	
12	7953	7483	-	hp				YifL; Predicted small periplasmic lipoprotein YifL (98,6/$2,8e^{-7}$)	hp

								Lysis_col; Lysis protein (97,41/0,00023)	
								Slp; Starvation-inducible outer membrane lipoprotein, 97,11/0,00057	
								COG5510; Predicted small secreted protein, (96,95/0,0014)	
13	8075	7950	-	ribonuclease [*Bacillus* phage Leo2]	$3e^{-17}$			hp	hp
14	8461	9303	+	scaffold protein [*Bacillus* virus Curly]	0.0			Mu-like_Pro; Mu-like prophage I protein (99,88/6,4e^{-21})	prohead protease
				scaffold protein [*Bacillus* virus Andromeda]	0.0			Phage_GPO; Phage capsid scaffolding protein (GPO) serine peptidase (99,57/4,1e^{-13})	
				scaffold protein [*Bacillus* virus Riggi]	$3e^{-170}$			proheadase_HK97; phage prohead protease, HK97 family (99,3/7,8e^{-11})	
								Peptidase_S78; *Caudovirus* prohead serine protease (99,14/1,2e^{-9})	
15	9325	9747	+	hp				COG5471; Predicted phage recombinase, RecA/RadA family (99,67/5,7e^{-15})	DUF2190 domain-containing protein
								COG5471; Predicted phage recombinase, RecA/RadA family (86,05/7,4)	
16	9767	10657	+	major capsid protein [*Bacillus* virus Glittering]	0.0			d,183,1,1 (A:104-383) Major capsid protein gp5 {Bacteriophage HK97 [TaxId: 37554]} (99,95/4,6e^{-27})	Major capsid protein
				major capsid protein [*Bacillus* phage Leo2]	0.0			major_cap_HK97; phage major capsid protein, HK97 family	

								(99,93/1,5e^{-24})	
				major capsid protein [*Bacillus* virus Riggi]	0.0			COG4653; Predicted phage phi-C31 gp36 major capsid-like protein (99,93/8,4e^{-25})	
17	10732	10953	+	hp				Ribosomal_L9_N; Ribosomal protein L9, N-terminal domain (95,15/0,039)	hp
								d,100,1,1 (A:1-52) Ribosomal protein L9 N-domain {*Bacillus stearothermophilus* [TaxId: 1422]} (93,69/0,15)	
								d,100,1,1 (I:1-55) Ribosomal protein L9 N-domain {*Thermus thermophilus* [TaxId: 274]} (91,77/0,35)	
								Packaging_FI; DNA packaging protein FI (90,85/0,42)	
18	10967	11359	+	head-to-tail adaptor [*Bacillus* phage 056SW001B]	4e^{-26}	DUF1320 (2-65)	2.42e^{-06}	gp436; Mu-like prophage protein gp36 (99,88/5e^{-21})	head completion protein
				head-to-tail adaptor [*Bacillus* phage 276BB001]	6e^{-26}	gp436 (2-122)	7.41e^{-03}	Phage_connect_1; Phage gp6-like head-tail connector protein (98,76/1,3e^{-7})	
				head-to-tail adaptor [*Bacillus* phage 019DV002]	1e^{-25}				
19	11364	11807	+	tail completion and head joining protein [*Bacillus* virus Riggi]	6e^{-72}	COG5005 (4-147)	9.42e^{-13}	Phage_tail_S; Phage virion morphogenesis family (99,85/5,7e^{-21})	tail completion protein
				tail completion and head joining protein [*Bacillus* virus Riggi]	6e^{-72}	Phage_tail_S (6-141)	4.59e^{-08}	COG5005; Mu-like prophage protein gpG, (99,8/2,6e^{-18})	
				head-to-tail stopper [*Bacillus* phage 276BB001]	2e^{-52}			tail_comp_S; phage virion morphogenesis (putative tail completion) protein, (99,79/2,7e^{-}	

								^{18})	
20	11804	12232	+	tail terminator [*Bacillus* phage 019DV002]	$4e^{-37}$			Phage_tail_U; Phage minor tail protein U (99,66/2,6e^{-14})	tail terminator protein
								Gp37; Gp37 protein (97,15/0,016)	
21	12244	12420	+	hp				hp	hp
22	12420	13358	+	major tail protein [*Bacillus* phage 019DV002]	$9e^{-106}$			DUF2001; Phage tail tube protein 98,49/b,106,1,3 (A:5-143) Phage-like element PBSX protein XkdM {*Bacillus subtilis* [TaxId: 1423]} (98,24/0,000014)	tail tube protein
								Phage_tail_2; Phage tail tube protein (93,86/2,2)	
23	13383	13724	+	tail assembly chaperone [*Bacillus* phage 019DV002]	$7e^{-19}$			Phage_TAC_4; Phage tail assembly chaperone (98,68/5,9e^{-7})	tail assembly chaperone
				tail assembly chaperone [*Bacillus* phage 019DV002]	$6e^{-18}$			Phage_TAC_1; Phage tail assembly chaperone (98,51/0,0000046)	
				tail assembly chaperone [*Bacillus* phage 276BB001]	$7e^{-14}$			Phage_TAC_14; Phage tail assembly chaperone protein, TAC (97,93/0,00022)	
24	13769	13966	+	aldolase/citrate lyase family protein [*Bacillus* virus Blastoid]	$1e^{-11}$			Phage_P2_GpE; Phage P2 GpE (98,53/3,9e^{-8})	tail assembly chaperone
				tail assembly chaperone [*Bacillus* phage 276BB001]	$1e^{-10}$			Phage_TAC_6; Phage tail assembly chaperone protein, TAC (96,41/0,013)	

				tail assembly chaperone [*Bacillus* phage 019DV002]	$4e^{-10}$			phage_TIGR02216; phage conserved hypothetical protein (95,51/0,055)	
25	13978	16296	+	tape measure protein [*Bacillus* virus Taylor]	0.0	HI1514 (62-292)	$2.10e^{-23}$	COG5283; Phage-related tail protein (99,26/0,000022)	tape measure protein
				tail length tape measure protein [*Bacillus* phage Carmen17]	$7e^{-21}$	tape_meas_nterm (129-203)	$2.58e^{-16}$	HI1514; Phage tail tape-measure protein, controls tail length (99,2/0,0000013)	
				tail length tape measure protein [*Bacillus* phage Wes44]	$7e^{-21}$	COG5412 (153-651)	$1.10e^{-11}$	YqbO; Phage-related minor tail protein (98,9/0,00031)	
26	16310	18805	+	tail fiber [*Bacillus* phage Leo2]	0.0			FN3; Fibronectin type 3 domain (99,74/2,$1e^{-14}$)	putative tail fiber protein
				tail fiber [*Bacillus* virus Blastoid]	0.0			Receptor mediating netrin-dependent axon guidance (99,7/4,$4e^{-13}$)	
				minor tail protein [*Bacillus* phage 276BB001]	0.0			Neural cell adhesion molecule L1 (99,5/1,$7e^{-10}$)	
27	18810	19109	+	minor tail protein [*Bacillus* phage 019DV002]	$1e^{-29}$			hp	hp
				minor tail protein [*Bacillus* phage 276BB001]	$3e^{-29}$				
28	19110	20777	+	putative tailspike, beta-helical glycoside hydrolase [*Bacillus* virus Andromeda]	0.0	Pectate_lyase_3 (44-183)	$6.75e^{-06}$	RR_plus_rpt_1; twin-arg-translocated uncharacterized repeat protein (99,94/5,$9e^{-22}$)	Pectate lyase superfamily protein
				putative tailspike, beta-helical glycoside [*Bacillus* phage Gemini]	0.0	NosD (355-553)	$5.33e^{-05}$	b,80,1,3 (A:) Rhamnogalacturonase A {Fungus (*Aspergillusaculeatus*) [TaxId: 5053]} (99,86/5,$4e^{-19}$)	

				tailspike [*Bacillus* virus Curly]	0.0			b,80,1,3 (A:) Rhamnogalacturonase A {Fungus (*Aspergillus aculeatus*) [TaxId: 5053]} (99,86/$5,4e^{-19}$)	
								b,80,1,3 (A:) Polygalacturonase {*Erwinia carotovora*, subsp, carotovora [TaxId: 554]} (99,82/$1,5e^{-16}$)	
29	20790	21740	+	minor tail protein [*Bacillus* phage 019DV002]	$8e^{-144}$			Phage_GPD; Phage late control gene D protein (GPD) (99,95/$1,7e^{-24}$)	Baseplate hub protein
				minor tail protein [*Bacillus* phage 276BB001]	$3e^{-143}$			COG4379; Mu-like prophage tail protein gpP (99,94/$7e^{-23}$)	
				minor tail protein [*Microbacterium* phage Alakazam]	$8e^{-21}$			VI_Rhs_Vgr; type VI secretion system Vgr family protein, (99,9/$8,4e^{-21}$)	
								Prophage_tail; Prophage endopeptidase tail (99,84/$1,9e^{-18}$)	
30	21737	21988	+	collagen triple helix repeat containing protein [*Bacillus* phage vB_BpsS-36]	$3e^{-18}$			gp45; Mu-like prophage protein gp45 (98,24/0,000022)	Tail spike protein
								Phage_base_V; Type VI secretion system, phage-baseplate injector (98/0,0001)	
								b,40,8,1 (A:6-129) Tail-associated lysozyme gp5, N-terminal domain {Bacteriophage T4 [TaxId: 10665]} (97,84/0,00033)	
31	22004	22420	+	phosphodiesterase [*Bacillus* virus Blastoid]	$6e^{-89}$	PRK12704 (13-134)	$2.49e^{-04}$	GvpP; Gas vesicle protein (98,44/0,000079)	putative holin
				phosphodiesterase [*Bacillus* virus Riggi]	$3e^{-86}$			RNase_Y; ribonuclease Y (98,29/0,00023)	
				transposase [*Bacillus* phage Leo2]	$2e^{-85}$	IATP (37-74)	$2.53e^{-03}$	EnvC; Septal ring factor EnvC, activator of murein hydrolases	

								AmiA and AmiB (97,960,0044)	
				holin [*Bacillus* phage 019DV002]	$2e^{-23}$	RNase_Y (15-131)	$5.13e^{-03}$	Holin_BhlA; BhlA holin family (97,78/0,0014)	
32	22432	23331	+	cell wall hydrolase/autolysin [Bac*i*llus virus Andromeda]	0.0	MurNAc-LAA (3-180)	$3.35e^{-62}$	c,56,5,6 (A:) N-acetylmuramoyl-L-alanine amidase CwlV {*Paenibacillus polymyxa* [TaxId: 1406]} (99,97/2,7e^{-28})	N-acetylmuramoyl-L-alanine amidase
						Amidase_3 (4-179)	$4.30e^{-55}$		
				cell wall hydrolase/autolysin [*Bacillus* virus Andromeda]	0.0	Ami_3 (64-179)	$3.29e^{-30}$	spore_cwlD; N-acetylmuramoyl-L-alanine amidase CwlD (99,95/3e^{-26})	
						spore_cwlD (2-179)	$9.79e^{-29}$		
				endolysin [*Bacillus* virus Glittering]	0.0	mltD (208-299)	$5.02e^{-23}$	c,56,5,6 (A:1-180) Endolysin Ply, catalytic domain {Bacteriophage Psa [TaxId: 171618]} (99,95/1,4e^{-25})	
						LysM (209-251)	$7.49e^{-16}$		
						spore_safA (211-250)	$2.03e^{-04}$		
33	23416	23625	+	holin [*Bacillus* virus Glittering]	$7e^{-42}$			Phage_holin_1; Bacteriophage holing (99,04/1,1e^{-9})	holin
								holin_phiLC3; holin, phage phi LC3 family (98,97/1,7e^{-9})	
34	23800	24165	+	hp				hp	hp
35	24338	25231	+	ssDNA binding protein [*Bacillus* phage 019DV002]	$2e^{-96}$			gp32; gp32 DNA binding protein like (99,88/1,8e^{-21})	Single-stranded DNA-binding protein
				phosphoesterase [*Geobacillus* phage GBK1]	$4e^{-57}$				
36	25237	25590	+	hp				hp	hp
37	25637	26860	+	helicase [*Bacillus* virus Curly]	0.0	DnaB (17-429)	$1.47e^{-48}$	DnaB; Replicative DNA helicase (100/1,6e^{-35})	DnaB-like replicative helicase
				helicase [*Bacillus* virus Curly]	0.0	PRK08840 (49-376)	$6.93e^{-24}$	DnaB; replicative DNA helicase, This model describes the helicase DnaB, a homohexameric protein	

								required for DNA replication (100/1,3e^{-34})	
				DNA helicase [*Bacillus* virus Glittering]	0.0	DnaB_C (201-376)	4.40e-20	phage_DnaB; phage replicative helicase, DnaB family, HK022 subfamily, (100/5,1e^{-33})	
38	26853	27863	+	primase [*Bacillus* virus Andromeda]	0.0	dnaG (7-313)	7.97e-32	dnaG; DNA primase, catalytic core, (100/1e^{-38})	DnaG-like primase
				primase [*Bacillus* virus Curly]	0.0	Toprim_2 (226-308)	3.07e-14	DnaG; DNA primase (bacterial type), (100/1,9e^{-35})	
				primase [*Bacillus* virus Curly]	0.0	3.07e-14 (36-87)	4.89e-06	TraI_TIGR; conjugative transfer relaxase protein TraI (99,94/8e^{-25})	
39	27860	28213	+	hp				hp	hp
40	28215	28553	+	transcriptional repressor [*Bacillus* virus Glittering]	8e^{-70}	DeoR (60-91)	7.98e-05	Sig70_famx3; RNA polymerase sigma-70 factor, TIGR02954 family, (98,54/3,5e^{-7})	RNA polymerase sigma-70 factor
				transcriptional regulator [*Bacillus* virus Riggi]	1e^{-62}	Fis (57-94)	5.43e-03	SigE-fam_strep; RNA polymerase sigma-70 factor, sigma-E family, (98,47/0,0000013)	
				hp		sigma70-ECF (63-96)	5.71e-03	Sig-70_gvs1; RNA polymerase sigma-70 factor, Rhodopirellula/Verrucomicrobium family (98,39/8,8e^{-7})	
41	28566	31655	+	DNA polymerase III alpha subunit [*Bacillus* virus Curly]	0.0	dnaE (6-1029)	0.0	DnaE; DNA polymerase III, alpha subunit (100/6,3e^{-171})	DNA polymerase III alpha subunit
						Polc (6-992)	0.0		
				DNA polymerase III alpha subunit [*Bacillus* virus Andromeda]	0.0	PHP_PolIIIA_DnaE3 (6-285)	1.37e-96	polc; DNA-directed DNA polymerase III (polc) (00/6,3e^{-158})	
						DNA_pol3_alpha (299-559)	2.00e-91		

				DNA polymerase III alpha subunit [*Bacillus* virus Andromeda]	0.0	POLIIIAc (9-71)	$4.76e^{-16}$	polC_Gram_pos; DNA polymerase III, alpha chain, Gram-positive type (100/4,6e^{-124})	
42	31658	32503	+	UvrD/REP helicase [*Bacillus* virus Curly]	0.0	PDDEXK_1 (10-262)	$1.94e^{-17}$	COG2887; RecB family exonuclease (99,96/2,6e^{-26})	RecB family exonuclease
				PD-(D/E)XK nuclease [*Bacillus* virus Glittering]	0.0			cas_AF1879; CRISPR-associated protein Cas4/Csa1, subtype I-A/APERN (99,91/9,5e^{-22})	
				RecB-like protein [*Bacillus* phage Leo2]	0.0	COG2887 (12-178)	$1.56e^{-05}$	AddB; ATP-dependent helicase/DNAse subunit B (99,9/4,1e^{-21})	
				CRISPR/Cas system associated protein [*Bacillus* virus Finn]	0.0			rexB_recomb; ATP-dependent nuclease subunit B (99,89/1,1e^{-20})	
43	32500	33006	+	endonuclease [*Bacillus* phage Leo2]	$6e^{-103}$	DUF1064 (11-110)	$5.12e^{-28}$	c,52,1,16 (A:7-168) TnsA endonuclease, N-terminal domain {*Escherichia coli* [TaxId: 562]} (99,47/1,2e^{-13})	Endonuclease
				Holliday junction resolvase [*Bacillus* virus Blastoid]	$1e^{-102}$			Phage_endo_I; Phage endonuclease I (99,09/5,8e^{-10})	
				restriction endonuclease-like protein [*Bacillus* phage Anath]	$4e^{-31}$			Tn7_Tnp_TnsA_N; TnsA endonuclease N terminal (98,23/0,0000016)	
44	33053	33733	+	RNA polymerase sigma-70 factor [*Bacillus* virus Curly]	$7e^{-168}$	sigma70-ECF (80-220)	$5.01e^{-15}$	spore_sigmaE; RNA polymerase sigma-E factor (99,95/9,5e^{-26})	RNA polymerase sigma-E factor
				RNA polymerase sigma-70 factor [*Bacillus* virus Andromeda]	$1e^{-163}$	RpoE (77-225)	$8.44e^{-08}$	SigW_bacill; RNA polymerase sigma-W factor, This sigma factor is restricted to certain lineages of the order Bacillales (99,94/1,8e^{-25})	

				RNA polymerase sigma-70 factor [*Bacillus* virus Eoghan]	6e^{-136}	Sigma70_r4 (169-218)	8.72e^{-07}	RpoE_Sigma70; RNA polymerase sigma factor RpoE (99,94/2,5e^{-25})	
45	33730	33981	+	hp				hp	hp
46	33974	34255	+	hp				hp	hp
47	34248	34481	+	hp				hp	hp
48	34481	35191	+	thymidylate synthase [*Bacillus* phage Leo2]	1e^{-169}	Thy1 (21-225)	1.59e^{-41}	d,207,1,1 (A:1-216) Thy1 homologue {Thermotoga maritima [TaxId: 2336]} (100/6e^{-36})	thymidylate synthase
				thymidylate synthase [*Bacillus* virus Glittering]	2e^{-158}	thyX (9-232)	2.25e^{-38}	thyX; thymidylate synthase, flavin-dependent (100/6,1e^{-34})	
				ThyX-like thymidylate synthase [*Streptomyces* phage Blueeyedbeauty]	3e^{-82}	ThyX (6-230)	1.36e^{-30}	Thy1; Thymidylate synthase complementing protein (100/3,4e^{-33})	
49	35293	35439	+	hp				POTRA_TamA_1; POTRA domain TamA domain 1 (62,95/7,3)	hp
50	35453	35806	+	cysteine-rich domain contining protein [*Bacillus* virus Glittering]	2e^{-81}	PRK14276 (84-113)	2.33e^{-04}	DnaJ_C; DnaJ C terminal domain (97,47/0,00098)	DnaJ domain-containing protein
				cysteine-rich zinc finger protein [*Bacillus* virus Riggi]	4e^{-75}	COG1107 (80-113)	1.23e^{-03}	Molecular chaperone (DnaJ superfamily) (97,31/0,0015)	
						DnaJ_CXXCXGXG (83-113)	8.83e^{-03}	DnaJ; DnaJ-class molecular chaperone with C-terminal Zn finger domain (97,19/0,0029)	
51	35971	36153	+	hp				FeoB; Fe2+ transport system protein B (80,91/20)	Prediction lipoprotein signal peptide
52	36150	36314	+	hp				hp	hp
53	36527	36841	+	hp				hp	hp

54	36838	37425	+	deoxynucleoside monophosphate kinase [*Bacillus* virus Curly]	$2e^{-131}$			Pmev_kin_anim; phosphomevalonate kinase, animal type (99,78/9,$5e^{-17}$)	kinase
				phosphate mevalonate kinase [*Bacillus* phage Leo2]	$9e^{-118}$			c,37,1,17 (A:) Gluconate kinase {*Escherichia coli* [TaxId: 562]} (99,72/1,$5e^{-15}$)	
				nucleoside kinase [*Bacillus* phage Anath]	$3e^{-34}$			c,37,1,1 (A:3-127,A:159-222) Adenylate kinase {Maize (Zea mays) [TaxId: 4577]} (99,7/$1e^{-15}$)	
				P-loop containing nucleoside triphosphate hydrolase [*Listeria* phage LP-026]	$5e^{-29}$			c,37,1,1 (A:) Deoxynucleoside monophosphate kinase {Bacteriophage T4 [TaxId: 10665]} 99,7/1,$2e^{-14}$	
55	37425	37769	+	hp				Syntaphilin; Golgi-localised syntaxin-1-binding clamp (94,04/0,41)	hp
								Golgin_A5; Golgin subfamily A member (5 93,85/2,4)	
								Synaptic vesicle protein EHS-1 and related EH domain proteins (92,19/3,8)	
56	37916	38725	+	serine-threonine kinase [*Bacillus* virus Andromeda]	0.0	STKc_CDK9 (60-187)	$2.32e^{-04}$	d,144,1,7 (A:) Protein kinase C, theta type {Human (*Homo sapiens*) [TaxId: 9606]} (100/1,$9e^{-33}$)	protein kinase
				serine-threonine kinase [*Bacillus* virus Curly]	$1e^{-162}$	Pkinase (59-187)	$5.29e^{-03}$	d,144,1,7 (A:) cAMP-dependent PK, catalytic subunit {Baker's yeast (*Saccharomyces cerevisiae*) [TaxId: 4932]} (100/2,$6e^{-33}$)	
				serine-threonine protein kinase [*Bacillus* phage Leo2]	$2e^{-155}$			d,144,1,7 (E:) cAMP-dependent PK, catalytic subunit {Mouse (*Mus musculus*) [TaxId: 10090]} (100/3,$4e^{-32}$)	
57	38716	39147	+	hp				hp	hp

58	39119	39535	+					zf-C2HCIx2C; Zinc-finger (96,99/0,0014)<br>phage_arpU; phage transcriptional regulator, ArpU family (63,71/6,1)	hp
59	39532	39648	+	hp				hp	hp
60	39650	39997	+	hp				hp	hp
61	39997	40230	+	hp				hp	hp
62	40232	40540	+	hp				hp	hp
63	40540	40971	+					CopC; Copper-binding protein CopC (methionine-rich) (98,26/0,00011)<br>YifL; Predicted small periplasmic lipoprotein YifL (97,75/0,000078)<br>YifL; Predicted small periplasmic lipoprotein YifL (97,75/0,000078)<br>LPAM_1; Prokaryotic membrane lipoprotein lipid attachment site (97,56/0,00015)	hp
64	40988	41125	+	hp				hp	hp
65	41227	41610	+	hp				DUF2582; Winged helix-turn-helix domain (DUF2582) (98,65/$8,9e^{-7}$)<br>COG2865; Predicted transcriptional regulator, contains HTH domain (98,55/0,0000018)<br>a,4,5,60 (A:86-162) Segregation and condensation protein B, ScpB {*Chlorobium tepidum* [TaxId: 1097]} (98,41/0,000015)	putative transcriptional regulator

								PaaX; PaaX-like protein (98,17/0,000048)	
								a,4,5,2 (A:2-72) LexA repressor, N-terminal DNA-binding domain {*Escherichia coli* [TaxId: 562]} (98,15/0,000088)	
								a,4,5,39 (A:) Penicillinase repressor BlaI {*Bacillus licheniformis* [TaxId: 1402]} (98,14/0,00011)	
66	41612	42037	+	hp				hp	hp
67	42255	43544	+	FtsK/SpoIIIE ATPase [*Bacillus* virus Curly]	0.0	FtsK (190-388)	$2.23e^{-22}$	T7SS_EccC_a; type VII secretion protein EccCa (99,86/$9e^{-20}$)	FtsK/SpoIIIE family protein
								FtsK; DNA segregation ATPase FtsK/SpoIIIE and related proteins (99,76/$1,8e^{-16}$)	
				FtsK/SpoIIIE family protein [*Bacillus* virus Riggi]	0.0	PRK10263 (182-379)	$4.25e^{-13}$	PvdE; ABC-type siderophore export system, fused ATPase and permease components (98,69/$6,4e^{-9}$)	
				FtsK-like DNA translocase [*Bacillus* phage 019DV002]	0.0	T7_EssCb_Firm (163-389)	$8.33e^{-10}$	Multidrug resistance-associated protein/mitoxantrone resistance protein, ABC superfamily (98,57/$4,4e^{-8}$)	
68	43807	44082	+	replication/relaxation protein [*Bacillus* virus Curly]	$5e^{-63}$			Replic_Relax; Replication-relaxation (98,96/$3,8e^{-9}$)	Replic_Relax superfamily protein
				plasmid replication-relaxation protein [*Bacillus* virus Riggi]	$1e^{-60}$			DpnII; DpnII restriction endonuclease (93,27/1,1)	
				plasmid replication-relaxation protein [*Bacillus* virus Blastoid]	$1e^{-60}$			capSnatchArena; RNA endonuclease, cap-snatching, arenavirus family (92,65/0,69)	

69	44095	44508	+	hp				TrbC; TrbC/VIRB2 family (98,95/1,6e^{-8})	TrbC/VIRB2 family protein
								VirB2; Type IV secretory pathway (98,47/0,0000064)	
								conj_TIGR03745; integrating conjugative element membrane protein, PFL_4702 family (98,17/0,00014)	
								Maff2; Maff2 family (97,51/0,0036)	
70	44579	44782	+	DNA binding domain protein [*Bacillus* phage 019DV002]	6e^{-25}	gatA (22-43)	6.87e^{-03}	Arc; Arc-like DNA binding domain (96,23/0,033)	DNA binding domain protein
								antidote_CC2985; putative addiction module antidote protein, CC2985 family (95,93/0,045)	
				hp				COG4710; Predicted DNA-binding protein with an HTH domain 95,88/0,12	
								a,43,1,1 (A:) Arc repressor {*Salmonella* bacteriophage P22 [TaxId: 10754]} (94,95/0,16)	
71	45289	44801	-	hp				hp	hp
72	46030	45437	-	DNA polymerase III epsilon subunit [*Bacillus* virus Curly]	8e^{-142}	DEDDh (4-157)	3.95e^{-37}	PolC; DNA polymerase III, alpha subunit (gram-positive type) (99,87/6,4e^{-20})	DEDDh exonuclease
						EXOIII (1-162)	8.53e^{-26}		
				DNA polymerase III epsilon subunit [*Bacillus* virus Glittering]	2e^{-137}	DnaQ (1-162)	9.01e^{-26}	dnaQ_proteo; DNA polymerase III, epsilon subunit, Proteobacterial (99,85/7,1e^{-19})	
						RNase_T (4-156)	5.32e^{-25}		
				DEDDh 3'-5' exonuclease [*Paenibacillus* phage Scottie]	3e^{-35}	polC_Gram_pos (2-167)	3.02e^{-24}	dinG_rel; DnaQ family exonuclease/DinG family helicase, putative (99,82/7,1e^{-18})	
73	46767	46030	-	peptidase [*Bacillus*	2e^{-50}			ImmA; Zn-dependent peptidase	peptidase

				phage Leo2]				ImmA, M78 family (98,82/7,8e^{-8})	
								Peptidase_M78; IrrE N-terminal-like domain (98,69/2,3e^{-7})	
				zinc-dependent metalloprotease [*Bacillus* virus Blastoid]	9e^{-49}			COG3800; Predicted transcriptional regulator (98,39/0,0000038)	
								MPTase-PolyVal; Metallopeptidase superfamily domain (98,05/0,000016)	
74	46995	46768	-					SlyX; SlyX (92,79/1,4)	hp
								GTPase-activating protein 92,01/2,1	
								SlyX; Uncharacterized coiled-coil protein SlyX (sensitive to lysis X) (89,6/3,7)	
75	47355	47837	+	Holliday junction resolvase [*Bacillus* virus Glittering]	5e^{-111}	PHA00159 (58-129)	4.46e^{-06}	c,52,1,17 (A:) Endonuclease I (Holliday junction resolvase) {Bacteriophage T7 [TaxId: 10760]} (99,43/1,8e^{-12})	holliday junction resolvase
				T7-like endonuclease [*Bacillus* virus Curly]	2e^{-110}	Phage_endo_I (58-129)	8.84e^{-04}	Phage_endo_I; Phage endonuclease I (98,73/1,4e^{-7})	
				endonuclease [*Bacillus* phage Leo2]	7e^{-109}			Vsr; DNA mismatch endonuclease Vsr (98,08/0,000012)	
76	47884	48045	+	hp				hp	hp
77	48046	48210	+	hp				FlaG; Uncharacterized conserved protein, FlaG/YvyC family (99,76/4,3e^{-18})	FlaG/YvyC family protein
								d,352,1,1 (A:2-109) Hypothetical protein YvyC {*Bacillus subtilis* [TaxId: 1423]} (99,76/3,7e^{-18})	
								FlaG; FlaG protein 99,74/7,6e^{-18}	
78	48210	48377	+	hp		nifE (5-42)	9.38e^{-03}	e,3,1,1 (A:328-668) Penicillin binding protein 2a (PBP2A), C-terminal domain {*Staphylococcus*	hp

								aureus [TaxId: 1280]} (90,99/0,53)	
								Activator-TraM; Transcriptional activator TraM (89,76/0,3)	
								XisI; XisI protein (88,95/1,5)	
								bioW; 6-carboxyhexanoate--CoA ligase, Alternate name: pimeloyl-CoA synthase, [Biosynthesis of cofactors, prosthetic groups, and carriers, Biotin] (87,93/1,2)	
79	48402	48554	+	hp				Transcription factor (contains Zn finger) (97,82/0,0000046)	putative transcription factor
								CpXC; CpXC protein (97,57/0,00023)	
								Transcription factor BLIMP-1/PRDI-BF1, contains C2H2-type Zn-finger and SET domains (97,45/0,000092)	
								Transcriptional repressor SALM (97,4/0,0000058)	
80	48556	48702	+	hp				hp	hp
81	48896	49570	+	putative terminase small subunit [*Bacillus* phage 056SW001B]	$1e^{-77}$			sm_term_P27; phage terminase, small subunit, putative, P27 family (89,28/3,6)	putative terminase small subunit
				putative terminase small subunit [*Bacillus* phage 019DV002]	$6e^{-76}$			Terminase_4; Phage terminase, small subunit (88,04/5,6)	
				putative terminase small subunit [*Bacillus* phage 276BB001]	$6e^{-76}$			COG3747; Phage terminase, small (86,89/9,2)	
				exopolyphosphatase domain protein	$3e^{-64}$				

				[*Geobacillus* phage GBK1]					

Table S2. Annotation of the Bacillus phage Novomoskovsk

ORF№	Start codon	Stop codon	Strand	Blast results		Conserved domains, Blast		Hhpred results	Annotation
				Name	E-val	Name, (region)	E-val	(Prob./E-val)	
1	65	739	+	Putative terminase small subunit [*Bacillus phage* 056SW001B]	1e-73			sm_term_P27; phage terminase, small subunit, putative, P27 family, 92,77/0,94<br>Terminase_4; Phage terminase, small subunit (91,46/2)<br>COG3747; Phage terminase, small subunit (90,62/2,5)	Terminase, small subunit
2	1292	1074	-	hp					hp
3	1756	1457	-	hp				g,37,1,1 (A:67-101) Zinc finger protein 295, ZNF295 (95,4/0,0066)	hp
4	2006	1749	-	hp					hp
5	2189	2031	-	hp				FixS; Cytochrome oxidase maturation protein cbb3-type (77,84/8,2)<br>ccoS; cytochrome oxidase maturation protein, cbb3-type, CcoS from Rhodobacter capsulatus has been shown essential for incorporation of redox-active prosthetic groups (heme, Cu) into cytochrome cbb (3) oxidase, (76,32/10)<br>CD225; Interferon-induced transmembrane protein	hp

								(72,77/6,3)	
								FixS; Cytochrome oxidase maturation protein, CcoS/FixS family [Posttranslational modification, protein turnover, chaperones] (75,87/11)	
6	2407	2195	-	hp					hp
7	2700	2404	-	hp					hp
8	3089	2832	-	hp					hp
9	3318	3091	-	hp					hp
10	3419	5104	+	Terminase [*Bacillus* virus Finn]	0.0	Terminase_GpA (52-314)	$1.14 \cdot 10^{-5}$	Terminase_GpA; Phage terminase large subunit (GpA) (100/1,5e$^{-31)}$	Terminase, large subunit
								Terminase_1; Phage Terminase (100/1,2e^{-31})	
								COG5323; Large terminase phage packaging protein (100/3e^{-30})	
11	5187	6506	+	Portal [*Bacillus* virus Riggi]	0.0	DUF935 (90-394)	$4.54 \cdot 10^{-21}$	gp29; Mu-like prophage protein gp29 100/4e^{-31}	Portal protein
				Portal protein [*Bacillus* virus Glittering]	0.0			portal_HK97; phage portal protein, HK97 family, (99,97/6,6e^{-28})	
				Portal protein [*Bacillus* virus Curly]	0.0	gp29 (93-397)	$4.56 \cdot 10^{-8}$	BeeE; Phage portal protein BeeE (99,96/4,1e^{-28})	
				Portal protein [*Bacillus* virus Blastoid]	0.0			Phage_portal; Phage portal protein (99,96/1,5e^{-27})	
12	6560	7462	+	Phage head morphogenesis protein [*Bacillus* virus Finn]	0.0	Phage_Mu_F (144-254)	$8.39 \cdot 10^{-15}$	phageSPP1_gp7; phage putative head morphogenesis protein, SPP1 gp7 family, (99,85/2,3e^{-20})	Minor head protein
				Minor head protein	0.0	PhageSPP1_gp7	$3.60 \cdot 10^{-14}$	Phage_min_cap2; Phage minor	

				[*Bacillus* virus Riggi]		(151-261)		capsid protein 2 (99,84/4,3e^{-19})	
				Minor head protein [*Bacillus* phage Leo2]	0.0	COG2369 (19-264)	$2.12 \cdot 10^{-3}$	Phage_Mu_F; Phage Mu protein F like protein (99,81/1e^{-18})	
13	7977	7495	-	hp				YifL; Predicted small periplasmic lipoprotein YifL (98,17/9,2e^{-6})	Putative signal peptide-containing protein
								COG5510; Predicted small secreted protein (97,12/0,001$^{)}$	
14	8096	7977	-	Ribonuclease [*Bacillus* phage Leo2]	3e^{-13}				hp
				Hypothetical protein Riggi_14 [*Bacillus* virus Riggi]	6e^{-15}				
15	8516	9352	+	Scaffold protein [*Bacillus* virus Finn]	2e^{-175}			Mu-like_Pro; Mu-like prophage I protein (99,9/1,4e^{-21})	Prohead protease
				Scaffold protein [*Bacillus* virus Riggi]	1e^{-172}			COG4388; Mu-like prophage I protein [Mobilome: prophages, transposons], (99,87/1,8e^{-20})	
				Scaffold protein [*Bacillus virus* Curly]	8e^{-170}			Phage_GPO; Phage capsid scaffolding protein (GPO) serine peptidase (99,52/1,6e^{-13})	
				Putative protease I [Prokaryotic dsDNA virus sp.]	2e^{-4}			proheadase_HK97; phage prohead protease, HK97 family, This model describes the prohead protease of HK97 and related phage, (99,32/8,4e^{-11})	
								COG3740; Phage head maturation protease [Mobilome: prophages, transposons], (99,15/2,6e^{-9})	

16	9373	9795	+	hp				COG5471; Predicted phage recombinase, RecA/RadA family (99,75/1,1e^{-16})	DUF2190 domain-containing protein
								DUF2190 (99,49/2,8·10^{-12})	
17	9814	10707	+	Major capsid protein [*Bacillus* phage Leo2]	0.0			Major capsid protein gp5 {Bacteriophage} HK97 (99,95/3,3e^{-26})	Major capsid protein
				Major capsid protein [*Bacillus* virus Glittering]	0.0			major_cap_HK97; phage major capsid protein, HK97 family, (99,93/3e^{-23})	
				Major capsid protein [*Bacillus* virus Riggi]	0.0			Phage_capsid; Phage capsid family (99,9/5,3e^{-23})	
18	10726	10995	+	hp				Rep-A_N; Replication factor-A protein 1, N-terminal domain (47,48/25)	hp
19	11070	11291	+	hp				Ribosomal_L9_N; Ribosomal protein L9, N-terminal domain (94,28/0,068)	hp
								d,100,1,1 (A:1-52) Ribosomal protein L9 N-domain {*Bacillus stearothermophilus*} (94,03/0,095)	
								Packaging_FI; DNA packaging protein FI (92,83/0,29)	
20	11305	11697	+	hp		DUF1320 (2-65)	2.18·10^{-5}	gp436; Mu-like prophage protein gp36 (99,83/4,3e^{-19})	head completion protein
						gp436 (2-74)	2.98·10^{-3}	Phage_connect_1; Phage gp6-like head-tail connector protein (98,58/0,0000011$^{)}$	

21	11886	11767	-	hp					hp
22	12029	12145	+	Virion morphogenesis protein [*Bacillus phage* Leo2]	$3e^{-20}$			COG5005; Mu-like prophage protein gpG [Mobilome: prophages, transposons], (98,95/3,8e^{-9})	tail completion protein
				Tail completion and head joining protein [*Bacillus virus* Riggi]	$9e^{-9}$			Phage_tail_S; Phage virion morphogenesis family (98,91/9e^{-10})	
				Head-to-tail stopper [*Bacillus phage* 276BB001]	$3e^{-6}$			Tail_comp_S; phage virion morphogenesis (putative tail completion) protein, This model describes protein S of phage P2, suggested experimentally to act in tail completion and stable head joining, and related proteins from a number of phage, (98,65/3,7e^{-8})	
				Tail completion protein [*Bacillus virus* Blastoid]	$5e^{-8}$			Phge_HK97_gp10; phage protein, HK97 gp10 family, This model represents an uncharacterized, highly divergent bacteriophage family (98,63/7,2e^{-8})	
23	12142	12576	+	hp				Phage_tail_U; Phage minor tail protein U (99,57/1,3e^{-13})	tail terminator protein
24	12588	12764	+	hp				Predicted translation initiation factor related to eIF-1A [Translation, ribosomal structure and biogenesis] (72,07/13)	hp
								eIF-1A; eukaryotic/archaeal initiation factor 1A, Recommended nomenclature: eIF-1A for eukaryotes, aIF-1A for Archaea (70,11/20)	

								b,40,4,5 (A:) Archaeal initiation factor-1a, aIF1a {*Methanococcus jannaschii* [TaxId: 2190]} (67,47/21)	
25	12764	13702	+	Major tail protein [*Bacillus* phage 019DV002]	$3e^{-104}$			Phage tail tube protein 98,54/0,000012	Tail tube protein
				Major tail protein [*Bacillus* phage 276BB001]	$4e^{-104}$			Phage-like element PBSX protein XkdM {*Bacillus subtilis* 98,35/0,000046	
								Phage_tail_2; Phage tail tube protein 9(5,08/1,1)	
26	13727	14068	+	hp				Phage_TAC_4; Phage tail assembly chaperone (98,52/0,0000022)	Tail assembly chaperone
								Phage_TAC_1; Phage tail assembly chaperone 98,37/0,000009	
								Phage_TAC_5; Phage XkdN-like tail assembly chaperone protein, TAC (87,48/4,1)	
27	14113	14319	+	hp					hp
28	14323	16641	+	Tape measure protein [*Bacillus* virus Eoghan]	0.0	HI1514 (62-292)	$6.47e^{-26}$	COG5412; Phage-related protein (99,78/2,3e^{-12})	Tape measure protein
				Tape measure protein [*Bacillus* virus Taylor]	0.0	tape_meas_nterm (129-203)	$1.29e^{-16}$	HI1514; Phage tail tape-measure protein, controls tail length (99,11/0,0000036)	
				Tape measure protein [*Bacillus* phage Leo2]	0.0	COG5412 (153-666)	$4.42e^{-12}$		
				Tape measure protein [*Bacillus* virus Curly]	0.0	PHA01351 (462-596)	$2.73e^{-4}$		
29	16655	19150	+	Tail fiber [*Bacillus* virus Blastoid]	0.0			Receptor mediating netrin-dependent axon guidance (99,94/7,3e^{-23})	Putative tail protein
				Tail fiber [*Bacillus*	0.0			FN3; Fibronectin type 3 domain	

				phage Leo2]				$(99,8/6,1e^{-16})$	
				Minor tail protein [*Bacillus* phage 276BB001]	0.0			Neural cell adhesion molecule L1 $(99,74/1,6e^{-15})$	
								Peripheral benzodiazepine receptor PRAX-1 $99,68/(1,6e^{-3})$	
								Adhesion-type protein $(99,61/2,1e^{-13})$	
30	19155	19454	+	Minor tail protein [*Bacillus* phage 019DV002]	$4e^{-28}$				hp
31	19454	21085	+	Putative tailspike, beta-helical glycoside [*Bacillus* virus Finn]	0.0	Beta_helix (370-524)	$3.48e^{-12}$	phage_tailspike_middle; N-terminal and middle domains of tailspike protein in *Acinrtobacter* bacteriophages $(99,23/3e^{-8})$	Putative tailspike
				Tailspike [*Bacillus* virus Riggi]	0.0	NosD_copper_fam (252-497)	$5.79e^{-7}$		
				Tailspike [*Bacillus* virus Blastoid]	0.0	NosD (264-425)	$6.25e^{-3}$		
32	21100	22050	+	Minor tail protein [*Bacillus* phage 019DV002]	$6e^{-148}$			COG4379 Mu-like prophage tail protein gpP $(99,96/5,7e^{-25})$	Baseplate hub protein and central tail fiber
								COG3501 VgrG $99,93/1,3e^{-22}$	
				Minor tail protein [*Bacillus* phage 276BB001]	$3e^{-147}$			Prophage_tail; Prophage endopeptidase tail $(99,84/6,4e^{-19})$	
				Minor tail protein [*Streptomyces* phage Zuko]	$7e^{-45}$				

33	22047	22640	+	Collagen triple helix repeat containing protein [*Bacillus* phage vB_BpsS-36]	7e^{-110}			b,21,1,3 (A:162-264) receptor binding protein, rbp, C-terminal domain {*Lactococcus lactis*} phage p2 (96,75/0,031)	Receptor-binding protein
								Caudo_bapla_RBP; Receptor-binding protein of phage tail base-plate *Siphoviridae*, head (87,3/12)	
34	22657	23073	+	Phosphodiesterase [*Bacillus* virus Riggi]	1e^{-90}	PRK12704 (13-136)	4.23e^{-5}	RNase_Y; ribonuclease Y (98,98 \,1,3e^{-7})	putative holin
				Transposase [*Bacillus* phage Leo2]	1e^{-87}	RNase_Y (15-136)	3.32e^{-3}	Holin_BhlA; BhlA holin family (98,94/3,8e^{-8})	
				Phosphodiesterase [*Bacillus* virus Blastoid]	8e^{-81}	IATP (37-80)	5.87e^{-3}	AtpF; FoF1-type ATP synthase, membrane subunit b or b' (97,38/0,024)	
35	23087	23986	+	Cell wall hydrolase/autolysin [*Bacillus* virus Eoghan]	0.0	MurNAc-LAA (3-180)	1.88e^{-65}	c,56,5,6 (A:) N-acetylmuramoyl-L-alanine amidase CwlV {Paenibacillus polymyxa} (99,97/2,2e^{-28})	N-acetylmuramoyl-L-alanine amidase
						Amidase_3 (4-179)	2.17e^{-57}		
						mltD (200-296)	1.94e^{-21}		
						LysM (209-251)	1.68e^{-15}		
						spore_safA (211-250)	5.15e^{-4}		
36	24072	24281	+	hp				Phage_holin_1; Bacteriophage holin (99,59/9,6e^{-15})	Holin
								Holin_phiLC3; holin, phage phi LC3 family, Phage proteins for bacterial lysis typically include a membrane-disrupting protein, or holin, and one or more cell wall	

								degrading enzymes that reach the cell wall because of holin action, (99,56/1,4e^{-14})	
								Holin_SPP1; holin, SPP1 family, This model represents one of more than 30 families of phage proteins, all lacking detectable homology with each other, known or believed to act as holins, (90,01/2,1)	
37	24458	24823	+	Holin [*Bacillus* virus Andromeda]	3e^{-66}			DUF5105; Domain of unknown function (90,98/2,9)	hp
				Holin [*Bacillus* virus Blastoid]	6e^{-62}				
				Holin [*Bacillus* virus Curly]	2e^{-61}				
				Holin [*Bacillus* virus Finn]	2e^{-57}				
38	24987	25880	+	ssDNA binding protein [*Bacillus* phage 019DV002]	2e^{-96}			gp32; gp32 DNA binding protein like (99,86/8,5e^{-21})	Single-stranded DNA-binding protein
				ssDNA binding protein [*Bacillus* phage 056SW001B]	5e $^{-96}$				
39	25886	26239	+	hp					hp
40	26268	27509	+	Helicase [*Bacillus* virus Finn]	0.0	DnaB (21-433)	3.53e^{-50}	DnaB; Replicative DNA helicase (100/9,2e^{-36})	DnaB-like replicative helicase
						PRK07773 (21-379)	4.66e^{-22}		
						DnaB_C (205-380)	3.58e^{-20}		
41	27502	28512	+	DNA primase [*Bacillus* virus Riggi]	0.0	dnaG (3-314)	5.05e^{-32}	dnaG; DNA primase, catalytic core (100/2,7e^{-39})	DnaG-like primase
						Toprim_2 (212-294)	7.14e^{-14}		

						TOPRIM_DnaG _primases (212-285)	$2.15e^{-12}$		
						ZnF_CHCC (22-73)	$2.60e^{-6}$		
42	28509	28871	+	Putative 60 kDa chaperonin [Prokaryotic dsDNA virus sp.]	1.2				hp
43	28879	29199	+	Transcriptional regulator [*Bacillus* virus Riggi]	$2e^{-75}$	DeoR (57-88)	$1.66e^{-3}$	COG5606; Predicted DNA-binding protein, XRE-type HTH domain (97,56/0,00094)	Putative transcriptional regulator
44	29212	32292	+	DNA polymerase III [*Bacillus* virus Riggi]	0.0	dnaE (6-1026)	0		DNA polymerase III alpha subunit
				DNA polymerase III alpha subunit [*Bacillus* virus Finn]		polc (6-989)	0		
						PHP_PolIIIA_DnaE3 (6-285)	$3.49e^{-95}$		
						DNA_pol3_alpha (297-559)	$4.86e^{-92}$		
						POLIIIAc (9-71)	$2.31e^{-16}$		
45	32295	33140	+	PD-(D/E)XK nuclease [*Bacillus* virus Riggi]	0.0	PDDEXK_1 (10-196)	$5.16e^{-17}$	COG2887; RecB family exonuclease (99,96/2,3e^{-26})	RecB family exonuclease
						COG2887 (12-178)	$1.38e^{-4}$		
46	33137	33646	+	Endonuclease [*Bacillus* phage Leo2]	$5e^{-109}$	DUF1064 (11-110)	$1.04e^{-28}$	DUF1064 (99,67/2e^{-14})	Endonuclease
								TnsA endonuclease, N-terminal domain (99,47/2,1e^{-13})	
								Phage_endo_I (99,08/1,2e^{-9})	
47	33643	33915	+	hp		TM1586_NiRdase (7-77)	$3.15e^{-3}$	TA0956 ; *Thermoplasma acidophilum* protein TA0956	hp

						Frm2-like (7-66)	$6.52e^{-3}$	(92,18/0,55)<br>tRNA-Thr_ED ; Archaea-specific editing domain of threonyl-tRNA synthetase (91,98/2)	
48	33971	34651	+	RNA polymerase sigma 70 factor [*Bacillus* virus Riggi]	$8e^{-164}$	sigma70-ECF (80-220)<br>Sigma70_r4 (169-218)<br>RpoE (79-224)<br>PRK09647 (79-221)	$6.35e^{-16}$<br>$8.79e^{-8}$<br>$1.94e^{-7}$<br>$1.75e^{-4}$	spore_sigmaE; RNA polymerase sigma-E factor, Members of this family comprise the Firmicutes lineage endospore formation-specific sigma factor SigE, also called SpoIIGB and sigma (29, 99,95/4,$1e^{-26}$)	RNA polymerase sigma factor
49	34648	34899	+	hp					hp
50	34899	35609	+	Thymidylate synthase [*Bacillus* virus Blastoid]	$5e^{-168}$	thyX (9-232)<br>Thy1 (21-225)	$3.98e^{-41}$<br>$2.57e^{-40}$	thyX; thymidylate synthase, flavin-dependent (100/1,$4e^{-33}$)<br>Thy1; Thymidylate synthase complementing protein (100/3,$7e^{-33}$)	Thymidylate synthase
51	35712	36065	+	Cysteine-rich zinc finger protein [*Bacillus* virus Riggi]	$5e^{-82}$			g,54,1,1 (A:) Cysteine-rich domain of the chaperone protein DnaJ (98,13/0,000026)	DnaJ domain-containing protein
52	36062	36229	+	Protease [Human immunodeficiency virus 1]	0.72			PepSY_TM; PepSY-associated TM region 75,89/20<br>PiuB; Uncharacterized iron-regulated membrane protein [Function unknown] (68,96/35)<br>Predicted membrane protein, contains DoH and Cytochrome b-561/ferric reductase transmembrane domains [Signal transduction mechanisms]	hp

								(63,6/45)	
53	36230	36412	+	hp				Secretory carrier membrane protein [Intracellular trafficking, secretion, and vesicular transport] (75,88/19)<br>Phage_holin_3_5; Bacteriophage holin Hol, superfamily III (61,84/41)<br>FeoB; Fe2+ transport system protein B [Inorganic ion transport and metabolism] (61,62/68)	hp
54	36409	36585	+	DNA binding protein [*Bacillus* virus Blastoid]	$4e^{-26}$				hp
55	36798	37112	+	hp					hp
56	37113	37697	+	Deoxynucleoside monophosphate kinase [*Bacillus* virus Finn]<br>Phosphate mevalonate kinase [*Bacillus* phage Leo2]	$4e^{-143}$<br>$3e^{-124}$	PRK01184 (15-193)<br>P-mevalo_kinase (17-122)	$1.95e^{-6}$<br>$2.43e^{-6}$	Deoxynucleoside monophosphate kinase Bacteriophage T4 (99,85/2,3e^{-19})<br>Pmev_kin_anim; phosphomevalonate kinase, animal type, (99,85/5e^{-19})	Kinase
57	37697	38017	+	hp					hp
58	38080	38973	+	Serine/threonine kinase [*Bacillus virus* Riggi]	0.0	STKc_CK1 (88-149)	$5.59e^{-3}$	d,144,1,7 (A:) Protein kinase C, theta type {Human (*Homo sapiens*) [TaxId: 9606]} (99,97/2,3e^{-29})	Serine/threonine kinase
59	38970	39401	+	hp				BrnT_toxin; Ribonuclease toxin, BrnT, of type II toxin-antitoxin system (98,54,0,0000021)	hp

								flg_new; flagellar operon protein (97,96,0,000096)	
60	39373	39789	+	hp				zf-C2HCIx2C; Zinc-finger 97,1,0,0015	hp
61	39786	39917	+	hp				YycI; Two-component signal transduction system YycFG, regulatory protein YycI [Signal transduction mechanisms], (69,16/3,9)<br>Uncharacterized conserved protein, contains TBC domain [Signal transduction mechanisms, General function prediction only] (57,65/26)	hp
62	39904	40248	+	hp					hp
63	40245	40478	+	hp					hp
64	40480	40791	+	hp		N2227 (26-70)	7.92e^{-03}		hp
65	40791	41222	+	hp				YifL; Predicted small periplasmic lipoprotein YifL (98,07/0,0000075)	Predicted signal peptide-containing protein
66	41224	41376	+	hp				SPC12; Microsomal signal peptidase 12 kDa subunit (SPC12) (71,08/18)<br>Polytopic membrane protein Prominin [General function prediction only] (68,55/17)	hp
67	41470	41901	+	hp					hp
68	42119	43402	+	FtsK/SpoIIIE family	0.0	FtsK (190-436)	1.55e^{-22}	T7SS_EccC_a; type VII	FtsK/SpoIIIE-

				protein [*Bacillus* virus Riggi]				secretion protein EccCa (99,87/2,8e-^{20})	family protein
						FtsK_SpoIIIE (191-338)	$3.25e^{-12}$	FtsK; DNA segregation ATPase FtsK/SpoIIIE and related proteins (99,76/1,5e^{-16})	
						PRK10263 (189-379)	$7.78e^{-12}$		
						T7_EssCb_Firm (163-389)	$2.42e^{-9}$		
69	43665	43940	+	Replication/relaxation protein [*Bacillus* phage Leo2]	$9e^{-63}$			Replic_Relax; Replication-relaxation (98,97/3,5e^{-9})	Replic_Relax superfamily protein
70	43953	44366	+	hp				TrbC; TrbC/VIRB2 family (99,02/5e^{-9})	TrbC/VIRB2 family protein
								VirB2; Type IV secretory pathway, VirB2 components (pilins) (98,21/0,000039)	
71	44441	44644	+	Hypothetical protein Riggi_69 [*Bacillus* virus Riggi]	$2e^{-42}$			Arc; Arc-like DNA binding domain (97,97/0,000057)	Putative ParG-like protein
				Hypothetical protein ANDROMEDA_70 [*Bacillus* virus Andromeda]	$2e^{-42}$			a,43,1,1 (A:) Arc repressor {*Salmonella bacteriophage* P22 [TaxId: 10754]} (97,84/0,00011)	
				DNA binding domain protein [*Bacillus phage* 019DV002]	$4e^{-25}$	gatA (22-43)	$2.83e^{-03}$	a,43,1,1 (A:) Mnt repressor {*Salmonella bacteriophage* P22 [TaxId: 10754]} (97,75/0,00039)	
				DNA binding protein [*Bacillus phage* 035JT001]	$2e^{-5}$			RHH_7; Transcriptional regulator, RHH-like, CopG (97,69/0,0002)	

				DNA binding protein [*Bacillus phage* 022DV001]	$3e^{-5}$			a,43,1,3 (A:) Plasmid partition protein ParG {*Salmonella enterica* [TaxId: 28901]} (97,27/0,0021)	
72	45155	44664	-	hp					hp
73	45914	45318	-	DNA polymerase III [*Bacillus* phage Leo2]	$3e^{-120}$	DEDDh (5-158)	$1.94e^{-38}$	PolC; DNA polymerase III, alpha subunit (gram-positive type) (99,86/1,4e^{-19})	DEDDh exonuclease
						EXOIII (2-163)	$2.45e^{-28}$	Predicted exonuclease (99,82/4,5e^{-17})	
						PolC (3-168)	$2.08e^{-26}$		
						RNase_T (5-157)	$3.22e^{-26}$		
74	46624	45911	-	Zinc-dependent metalloprotease [*Bacillus* virus Blastoid]	$1e^{-47}$			ArdC; Antirestriction protein ArdC (99,03/3,3e^{-9})	Putative metallopeptidase
				Peptidase [*Bacillus* phage Leo2]	$2e^{-35}$			MPTase-PolyVal; Metallopeptidase superfamily domain (99,02/4,3e^{-9})	
								ImmA; Zn-dependent peptidase ImmA, M78 family (98,71/6,1e^{-8})	
75	46906	46625	-	hp					hp
76	47212	47694	+	Holliday junction resolvase [*Bacillus* virus Riggi]	$3e^{-112}$	PHA00159 (59-129)	$2.03e^{-05}$	Endonuclease I (Holliday junction resolvase) (99,12/1,2e^{-9})	Holliday junction resolvase

				T7-like endonuclease [*Bacillus* virus Finn]	$7e^{-112}$	Phage_endo_I (59-129)	$2.59e^{-03}$	Very short patch repair (VSR) endonuclease (98,98/3,$3e^{-9}$)	
77	47698	47844	+	hp				b,34,1,1 (A:189-235) Biotin--[acetyl-CoA-carboxylase] ligase C-terminal domain {*Pyrococcus horikoshii* [TaxId: 53953]} (75,62/7,5)	hp
78	47834	47989	+	hp				e,3,1,1 (A:328-668) Penicillin binding protein 2a (PBP2A), C-terminal domain {*Staphylococcus aureus* [TaxId: 1280]} (92,85,0,3)<br>CheD; Chemotaxis receptor (MCP) glutamine deamidase CheD [Cell motility, Signal transduction mechanisms], (86,32/1,9)<br>CheD ; CheD chemotactic sensory transduction (85,49/3)<br>e,3,1,1 (A:293-692) Penicillin-binding protein 2, PBP2 {*Staphylococcus aureus* [TaxId: 1280]} (85,24/2,5)	hp
79	48019	48171	+	hp				Transcription factor (contains Zn finger) [Transcription] (97,87/0,0000064)<br>CpXC; CpXC protein (97,66/0,000054)<br>Transcription factor BLIMP-1/PRDI-BF1, contains C2H2-type Zn-finger and SET domains [Transcription] (97,34/0,00009)	Putative DNA-binding protein

								Transcriptional repressor SALM [Transcription] (97,3/0,0000097)	
80	48173	48319	+	hp					hp
81	48492	49166	+	Putative terminase small subunit [*Bacillus* phage 056SW001B]	1e^{-73}			sm_term_P27; phage terminase, small subunit, (92,77/0,94)<br>Terminase_4; Phage terminase, small subunit (91,46/2)<br>COG3747; Phage terminase, small subunit (90,62/2,5)	Terminase, small subunit

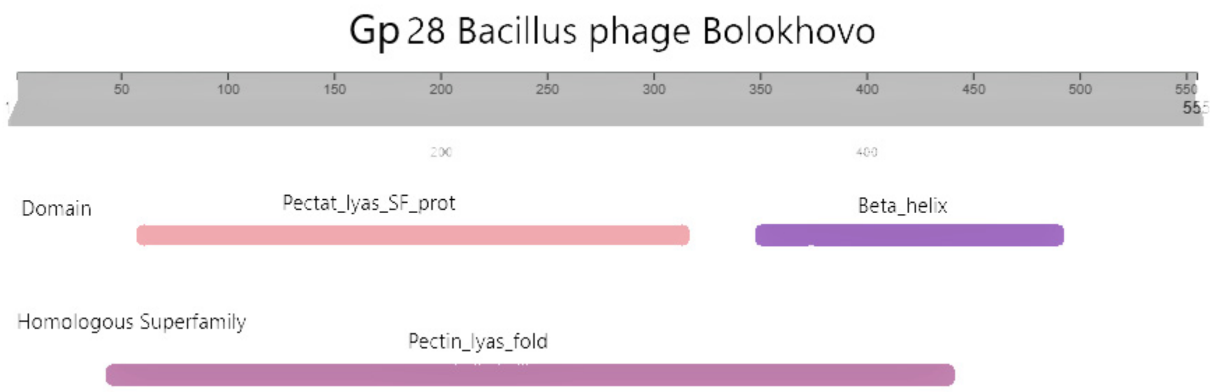

Figure S1. Domain organization of the depolymerase Gp28 of Bolokhovo

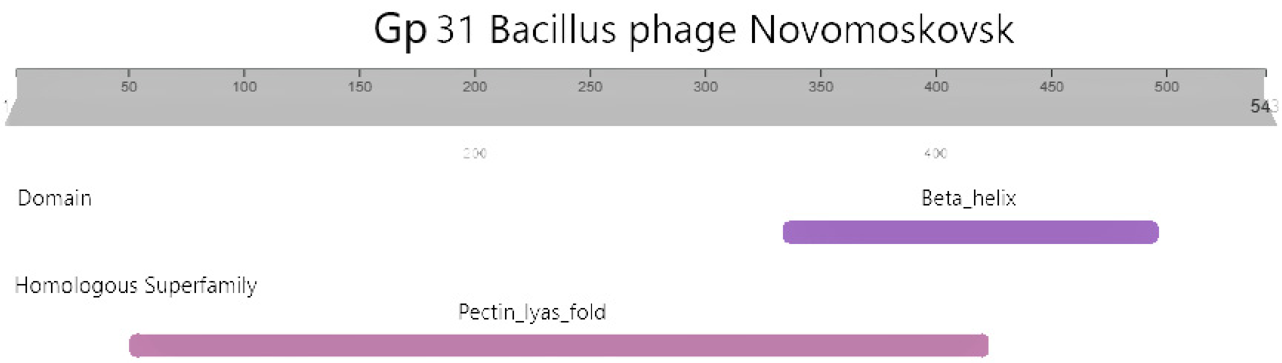

Figure S2. Domain organization of the depolymerase Gp31 of Novomoskovsk
